# Chronic Ethanol Consumption Alters Presynaptic Regulation of Dorsal Striatal Dopamine Release in C57BL/6J Mice

**DOI:** 10.3390/ijms231910994

**Published:** 2022-09-20

**Authors:** Armando G. Salinas, Jacob A. Nadel, Yolanda Mateo, Thanh Huynh, Shana M. Augustin, Karel Pacak, David M. Lovinger

**Affiliations:** 1 Laboratory for Integrative Neuroscience, Division of Clinical and Biomedical Research, National Institute on Alcohol Abuse and Alcoholism, National Institutes of Health, Bethesda, MD 20852, USA; 2 Department of Pharmacology, Toxicology & Neuroscience, Louisiana State University Health Sciences Center—Shreveport, Shreveport, LA 71103, USA; 3 Section on Medical Neuroendocrinology, The Eunice Kennedy Shriver National Institute of Child Health and Human Development, National Institutes of Health, Bethesda, MD 20892, USA; 4 Department of Pharmacology, Northwestern University Feinberg School of Medicine, Chicago, IL 60611, USA

**Keywords:** acetylcholine, dopamine, alcohol use disorder, addiction

## Abstract

Alcohol use disorder (AUD) is characterized by escalating alcohol consumption, preoccupation with alcohol, and continued alcohol consumption despite adverse consequences. Dopamine has been implicated in neural and behavioral processes involved in reward and reinforcement and is a critical neurotransmitter in AUD. Clinical and preclinical research has shown that long-term ethanol exposure can alter dopamine release, though most of this work has focused on nucleus accumbens (NAc). Like the NAc, the dorsal striatum (DS) is implicated in neural and behavioral processes in AUD. However, little work has examined chronic ethanol effects on DS dopamine dynamics. Therefore, we examined the effect of ethanol consumption and withdrawal on dopamine release and its presynaptic regulation with fast-scan cyclic voltammetry in C57BL/6J mice. We found that one month of ethanol consumption did not alter maximal dopamine release or dopamine tissue content. However, we did find that D2 dopamine autoreceptors were sensitized. We also found a decrease in cholinergic control of dopamine release via β2-containing nAChRs on dopamine axons. Interestingly, both effects were reversed following withdrawal, raising the possibility that some of the neuroadaptations in AUD might be reversible in abstinence. Altogether, this work elucidates some of the chronic alcohol-induced neurobiological dysfunctions in the dopamine system.

## 1. Introduction

Alcohol use disorder (AUD) is a broad condition with diagnostic criteria including, but not limited to, escalation of alcohol seeking and consumption, preoccupation with alcohol, and continued alcohol consumption despite adverse personal or professional consequences [1]. The financial consequences of AUD exceed USD 240 billion in annual losses in the US alone [2]. Furthermore, more than 90,000 deaths annually are attributed to AUD either directly or indirectly [3]. Despite the prevalence of AUD, treatments are limited and each with limited efficacy. Thus, a greater understanding of the neurobiology contributing to AUD and associated brain changes is essential to developing novel therapeutic strategies.

The dorsal striatum (DS), or caudate nucleus and putamen in primates, is a brain region implicated in movement and motor control, learning and memory, habit formation, and appetitive behaviors, including alcohol and substance use disorders [4]. Within the DS, the neuromodulator dopamine, which can operate on both phasic (seconds to minutes) or “tonic” (hours) time scales, is essential for several neurobiological processes including movement and synaptic plasticity. In humans, chronic ethanol consumption leads to dysregulation of dopamine signaling which is thought to contribute to AUD [5,6,7]. Similarly, in nonhuman primates trained to self-administer ethanol several adaptations in phasic striatal dopamine dynamics were observed [8,9,10,11,12,13]. In rodent AUD models using forced ethanol exposure (e.g., chronic intermittent ethanol vapor exposure or intragastric administration), phasic dopamine dynamics have also been shown to be differentially affected depending on the age of exposure, sex, species/strain, withdrawal status, and ethanol administration route [14,15,16,17,18,19,20,21]. Rodent studies using volitional ethanol consumption, which have greater face validity than forced ethanol exposure methods, to study ethanol induced changes in phasic striatal dopamine dynamics are less common [22,23].

Therefore, the current study examined the neurobiological consequences of chronic, daily ethanol consumption on phasic dopamine release and the presynaptic mechanisms regulating dopamine release, in the dorsal striatum. Thus, mice were exposed to a two-bottle choice drinking paradigm with one bottle containing water and the other containing a 10% ethanol solution. Mice were allowed to drink ethanol ad libitum for one day (acute consumption), one month (chronic consumption), or one month followed by one week of withdrawal. We then performed ex vivo fast-scan cyclic voltammetry (FSCV) and biochemical measurements of dorsal striatal dopamine. We found no significant differences in tonic dopamine tissue levels, or phasic dopamine release (at tonic or phasic firing frequencies) in our ethanol drinking mice. However, we did find differences in D2 dopamine autoreceptor and β2-containing nicotinic acetylcholine receptor (nAChR) regulation of dopamine release in our ethanol drinking mice. Interestingly, these neuroadaptations were reversed following one week of withdrawal from ethanol consumption. Altogether, these data suggest that chronic ethanol drinking induced neuroadaptations in D2 dopamine autoreceptors and β2-subunit containing nAChRs may be promising targets for future pharmacotherapeutic developments for AUD.

## 2. Results

### 2.1. Two-Bottle Choice Ethanol Consumption

**Figure 1:**
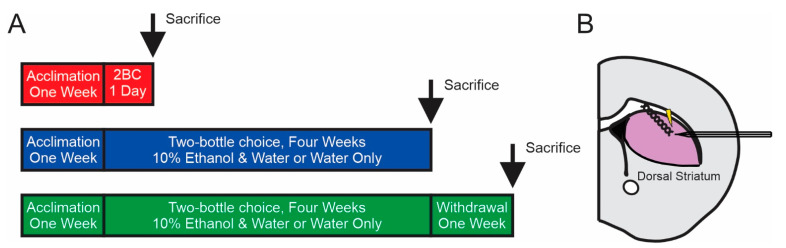
Schematic illustrating the experimental timeline (**A**) and fast-scan cyclic voltammetry recording area (**B**).

**Figure 2:**
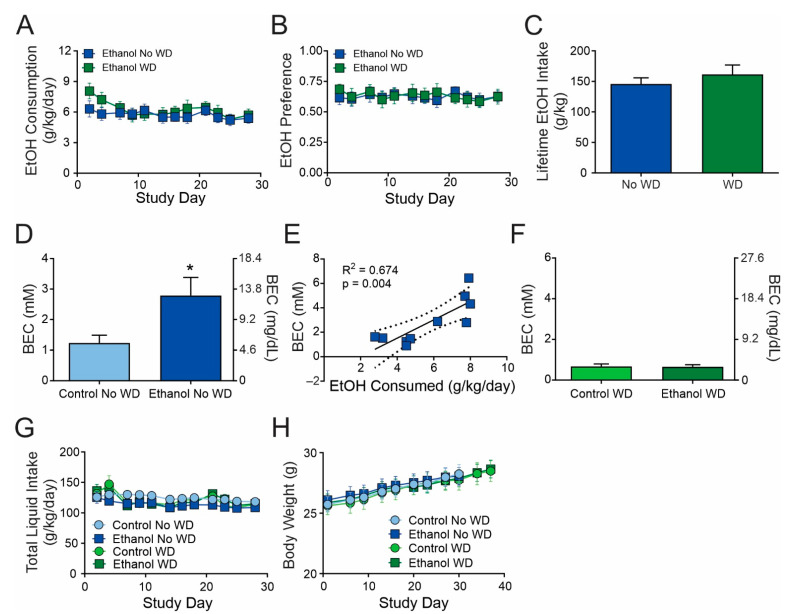
Ethanol drinking patterns were similar between groups. Ethanol consumption (**A**), preference (**B**), and lifetime ethanol intake (**C**) did not differ significantly between No WD and WD groups. Blood ethanol concentrations were elevated in the ethanol No WD group (**D**) and correlated with ethanol consumption (**E**). Blood ethanol concentrations were not elevated in the ethanol WD group (**F**). Total liquid intake (**G**) and body weights (**H**) did not differ between groups across the study. *n* = 7–10 mice per group. * *p* < 0.05.

Here, we examined the effects of acute (one day) and chronic (four weeks; No WD) ethanol consumption, as well as chronic ethanol consumption followed by one week of withdrawal (WD) on dopamine dynamics and mechanisms regulating its release (Figure 1A). Each group had a respective water control group that was treated identically to its ethanol drinking group except that they could consume only water. We found no significant differences in ethanol consumption (Figure 2A), ethanol preference (Figure 2B), or lifetime ethanol intake (Figure 2C) between the No WD and WD groups. Thus, any differences between these groups are likely due to withdrawal status and not any differences in ethanol exposure.

Blood ethanol concentrations were elevated in ethanol consuming mice (Figure 2D; t(17) = 2.308, *p* = 0.033) and correlated with ethanol intake (Figure 2E; r(8) = 0.821, *p* = 0.004). Neither total liquid intake (Figure 2G) nor body weights (Figure 2H) differed between the No WD and WD groups or their respective controls suggesting that the chronic ethanol consumption did not have any overt deleterious effects on mouse health.

### 2.2. Dopamine Release and Tissue Content Were Not Significantly Altered by Acute or Chronic Ethanol Consumption

**Figure 3:**
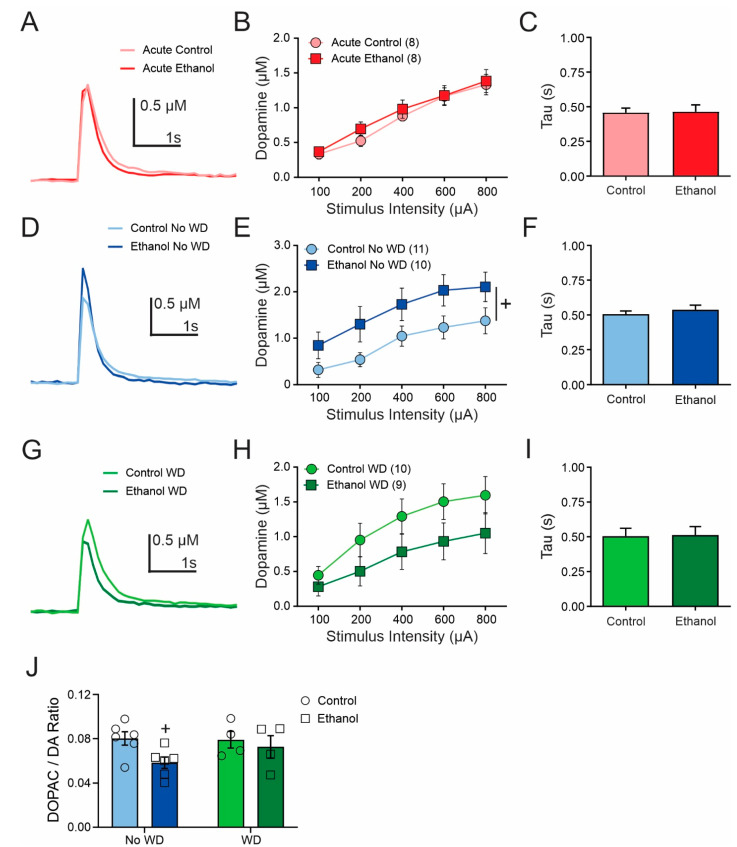
Two-bottle choice ethanol consumption did not significantly alter maximal dopamine release. One day of ethanol consumption (**A**,**B**) did not alter maximal dopamine release or dopamine transient decay rate (**C**). Four weeks of two-bottle choice drinking resulted in a trend towards increased maximal dopamine release (**D**,**E**) but no difference in dopamine transient decay rate (**F**). In the WD group, neither dopamine release (**G**,**H**) nor dopamine transient decay rate (**I**) differed from their respective controls. The DOPAC/DA ratio (**J**) trended towards a decrease in the ethanol No WD group compared to all the other groups. Number of slices indicated in parentheses from 8–10 mice per treatment group for FSCV experiments. *n* = 4–6 mice per group for DOPAC/DA ratios. + *p* < 0.10.

To examine potential differences in dopamine release, we performed ex vivo brain slice fast-scan cyclic voltammetry in dorsal striatal slices from mice of each group with varying stimulation intensities. In the acute drinking group and control, we found a main effect of stimulation intensity (F(1.343,18.80) = 62.54, *p* < 0.001) but no significant effect of treatment (F(1,14) = 0.317, *p* = 0.583) or stimulation intensity X treatment interaction (F(4,56) = 0.382, *p* = 0.821) (Figure 3A,B). To assess possible changes in dopamine uptake, we analyzed the dopamine transient decay rates and found no differences in these groups (Figure 3C). In the chronic ethanol drinking no WD group, we found a main effect of stimulation intensity (F(4,76) = 45.09, *p* < 0.001) but no significant main effect of treatment (F(1,19) = 3.562, *p* = 0.074) or stimulation intensity X treatment interaction (F(4.76) = 0.524, *p* = 0.718) (Figure 3D,E). Dopamine transient decay rates did not differ between treatment groups (Figure 3F). In the WD group, we found a main effect of stimulation intensity (F(4,68) = 46.63, *p* < 001) but no effect of treatment (F(1,17) = 1.97, *p* = 0.179) or stimulation intensity X treatment interaction (F(4,68) = 2.062, *p* = 0.095) (Figure 3G,H). Dopamine transient decay rates also did not differ between treatment groups (Figure 3I). Altogether, we found no significant effect of chronic two-bottle choice ethanol consumption on evoked dopamine release or uptake mechanisms.

To further examine chronic ethanol consumption effects on dorsal striatal dopamine, we also measured tonic tissue levels of dopamine and its metabolite DOPAC. We found a trend towards a significant effect of treatment group (F(1,16) = 4.02, *p* = 0.062) but no effects of withdrawal status (F(1,16) = 0.87, *p* = 0.365) or treatment X withdrawal status interaction (F(1,16) = 1.213, *p* = 0.287), on dopamine turnover as assessed with DOPAC/Dopamine ratios (Figure 3J).

### 2.3. Chronic Ethanol Consumption Increases D2 Dopamine Autoreceptor Sensitivity

**Figure 4:**
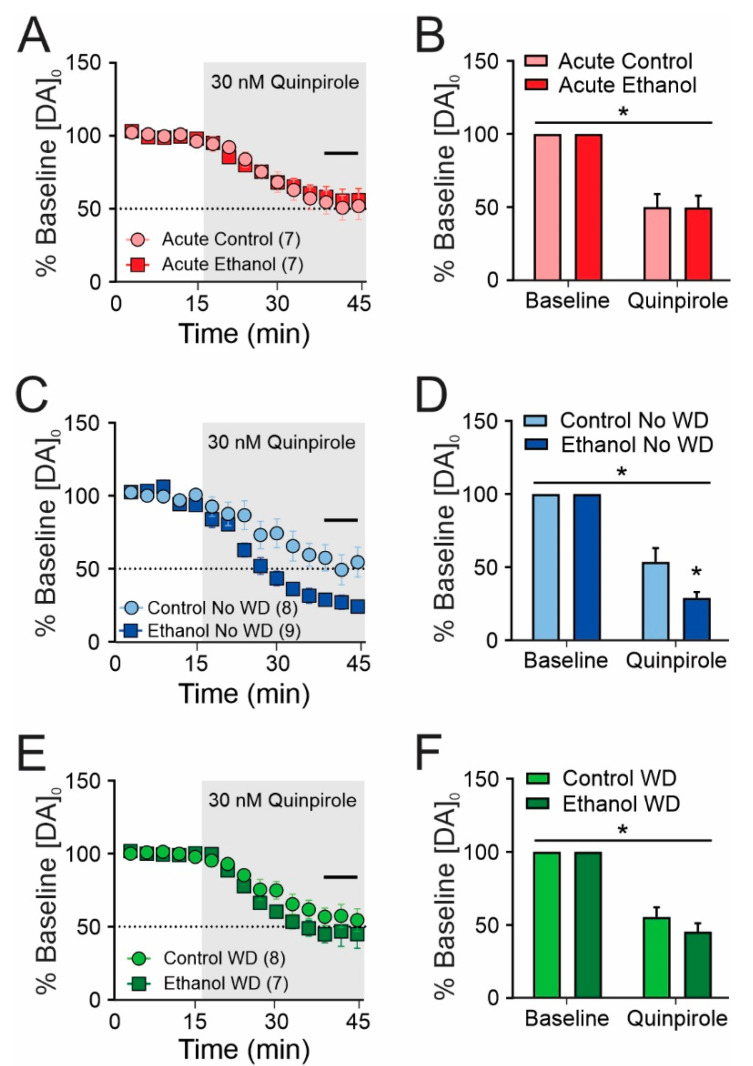
Chronic two-bottle choice ethanol consumption increases D2 dopamine autoreceptor sensitivity. One day of ethanol consumption (**A**,**B**) did not significantly alter D2 dopamine autoreceptor regulation of dopamine release. However, following four weeks of two bottle choice drinking, D2 dopamine autoreceptors were sensitized, resulting in a greater inhibition of dopamine release upon application of the D2-like dopamine receptor agonist, quinpirole (**C**,**D**), relative to control groups. This effect was absent in the WD group (**E**,**F**). The dotted lines in the time course graphs denote the 50% inhibition point. The number of slices for each group are indicated in parentheses and came from 7–9 mice per treatment group. * *p* < 0.05.

We next examined presynaptic mechanisms regulating dopamine release. The D2 dopamine autoreceptor is a Gi/o-coupled GPCR that will inhibit dopamine release upon activation. Exploiting this property, we applied the D2-like dopamine receptor agonist, quinpirole, at 30 nM (IC50 for inhibition of dopamine release in dorsal striatum) [24]. In all of the groups we found a significant main effect of quinpirole to inhibit dopamine release (Figure 4; Acute: (F(1,6) = 68.47, *p* < 0.001); Chronic No WD (F(1,15) = 140.6, *p* < 0.001); Chronic WD, (F(1,13) = 120.9, *p* < 0.001)). In the acute drinking group, we found no significant effects of treatment (F(1,6) < 0.001, *p* = 0.976) or a quinpirole X treatment interaction (F(1,6) < 0.001, *p* = 0.976) (Figure 4A,B). In contrast, in the chronic ethanol drinking no WD group, we found a treatment (F(1,15) = 6.193, *p* = 0.025) and quinpirole X treatment interaction (F(1,15) = 6.193, *p* = 0.025) such that quinpirole induced inhibition of dopamine release was sensitized relative to the control group (Figure 4C,D). These effects were reversed following one week of withdrawal as we found no significant effects of treatment (F(1,13) = 1.223, *p* = 0.289) or quinpirole X treatment interaction (F(1,13) = 1.223, *p* = 0.289) in the chronic ethanol drinking WD and control group (Figure 4E,F). In a separate mixed-effects model analysis, we compared the quinpirole effect between No WD and WD groups and found significant effects of quinpirole (F(1,15) = 249.4, *p* < 0.001), withdrawal status (F(1,15) = 6.741, *p* = 0.02), and a quinpirole X withdrawal status interaction (F(1,11) = 6.741, *p* = 0.025)

### 2.4. Chronic Ethanol Consumption Decreases nAChR Contribution to Dopamine Release

**Figure 5:**
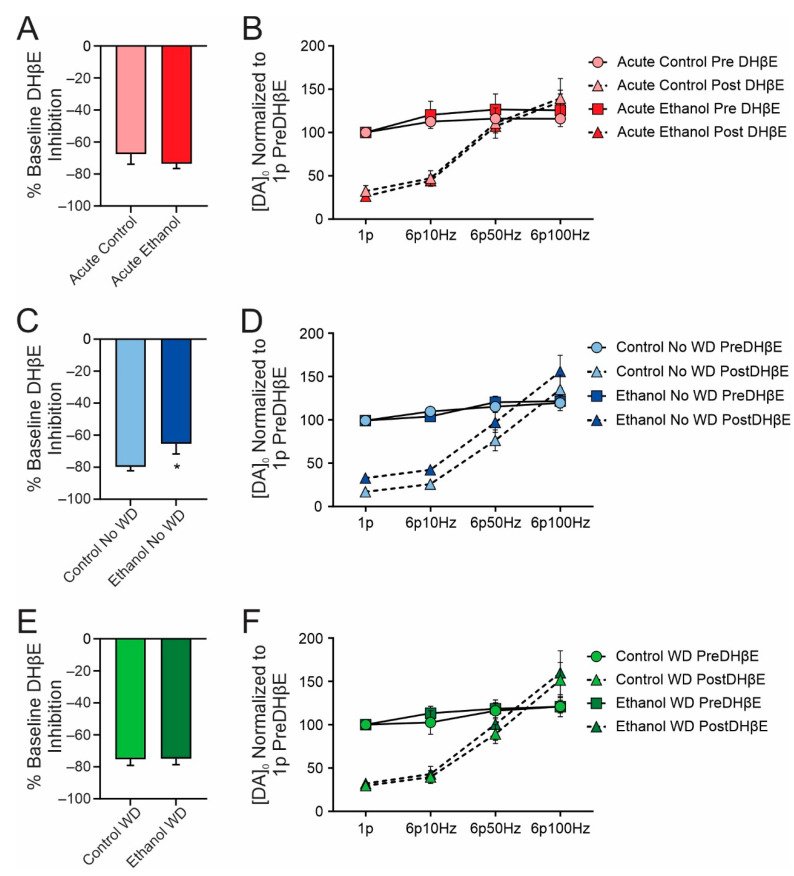
Chronic two-bottle choice ethanol consumption decreases the cholinergic contribution to dopamine release. One day of two-bottle choice (**A**) did not alter dopamine release regulation by the β2-containing nAChR antagonist, DHβE (1 µM), nor did it alter tonic or phasic dopamine release (**B**). In contrast, chronic two-bottle choice drinking resulted in a decreased inhibition of dopamine release by DHβE (**C**). Phasic release in this group was similar to controls (**D**). These effects were reversed following one week of WD (**E**,**F**). *n* = 5–9 slices per group from 5–7 mice per treatment group. * *p* < 0.05.

Activation of striatal cholinergic interneurons has been shown to facilitate dopamine release via activation of nAChRs located on dopamine axons [25,26,27,28]. Thus, pharmacological blockade of nAChRs will decrease electrically stimulated dopamine release. This blockade of nAChRs inhibited dopamine release equally between control and acute ethanol drinking mice (t(10) = 0.76, *p* = 0.465; Figure 5A). To examine potential differences in tonic and phasic release in this group, we applied electrical stimuli at different frequencies (10, 50, and 100 Hz) to emulate phasic dopamine release. We found significant effects of stimulation frequency (F(3,30) = 50.94, *p* < 0.001), DHβE application (F(1,10) = 15.88, *p* = 0.002), and a stimulation frequency X DHβE application interaction (F(3,30) = 58.20, *p* < 0.001) such that phasic stimulation in the presence of DHβE facilitated dopamine release (Figure 5B). In the chronic ethanol drinking no WD group, the nAChR inhibition was reduced relative to the control group with one pulse stimulations (t(13) = 2.248, *p* = 0.043) suggesting a decreased contribution of cholinergic signaling to dopamine release (Figure 5C). Following phasic stimulation, we found significant effects of stimulation frequency (F(3,45) = 69.87, *p* < 0.001), DHβE application (F(1,15) = 74.37, *p* < 0.001), a stimulation frequency X DHβE application (F(3,33) = 45.65, *p* < 0.001) and a treatment x DHβE application (F(1,33) = 4.2, *p* < 0.05) (Figure 5D). In the chronic ethanol drinking WD group, nAChR inhibition did not differ between ethanol drinking and control mice (t(20) = 0.058, *p* = 0.954) demonstrating that even one week of withdrawal can reverse some of the neurobiological adaptations to chronic ethanol drinking (Figure 5E). Similar to previous groups, following phasic stimulation, we found significant effects of stimulation frequency (F(3,39) = 51.21, *p* < 0.001), DHβE application (F(1,13) = 36.33, *p* = 0.002), and a stimulation frequency X DHβE application (F(3,39) = 52.76, *p* < 0.001) (Figure 5F). In a separate analysis, we examined the effect of withdrawal status on DHβE inhibition with a 3-way ANOVA (DHβE, withdrawal status, and ethanol treatment) and found significant effects of DHβE (F(1,27) = 916, *p* < 0.001), ethanol treatment (F(1,27) = 4.55, *p* = 0.042), and a DHβE X ethanol treatment interaction (F(1,27) = 4.55, *p* = 0.042).

## 3. Discussion

### 3.1. Summary of Findings

In the current study, we found that one month, but not one day, of two-bottle choice ethanol consumption led to several neuroadaptations in the presynaptic regulation of dopamine release. Specifically, we found that D2 dopamine autoreceptors were sensitized and exhibited a greater inhibitory control over dopamine release than in controls. We also found that the nAChR contribution to dopamine release was decreased in the ethanol consuming subjects relative to controls. Importantly, both of these effects were absent in the one-week withdrawal group, raising the possibility that long-term ethanol consumption-induced neuroadaptations might also be reversible in abstinent AUD patients.

### 3.2. Ethanol Drinking and Dopamine Release

Ethanol consumption, ethanol preference, and lifetime ethanol intake did not differ between the No WD and WD groups. Similarly, there were no differences in body weight or total liquid intake between groups across the study. Therefore, any group differences are likely due to differences in withdrawal status. In the current study, we utilized a 24 h access, two-bottle choice paradigm. This led to a stable level of ethanol intake in all mice across the study period. It is noteworthy that two-bottle choice drinking paradigms rarely achieve intoxicating BECs. Nonetheless, ethanol consumption positively correlated with BECs and resulted in significant neurobiological adaptations, suggesting that drinking to intoxication may not be necessary for neural changes to occur, only long-term consumption or exposure. Interestingly, however, these drinking-induced changes in the presynaptic regulation of dopamine release were plastic and reversed following one week of withdrawal. The implications of this are promising and offer hope for AUD patients. This also raises the question of whether more severe ethanol consumption or ethanol exposure might lead to more lasting neurobiological changes in brain function. For example, might the decreased nAChR contribution to dopamine release be permanent in subjects following one year of two bottle choice or subjects frequently exposed to intoxicating levels of ethanol (e.g., as observed in chronic intermittent ethanol vapor models)?

Several groups have investigated the effects of chronic ethanol treatment on dopamine release and uptake in the nucleus accumbens or ventral striatum and found changes in either dopamine release or uptake. Most of these studies used chronic intermittent ethanol vapor exposure in mice and found decreases in dopamine release and increases in dopamine uptake rates [15,16,18,29]. Other studies have found no change [14,17] or increases in dopamine release [22]. In the current study, we found no significant group differences in evoked dopamine release or uptake. However, we did note a trend towards increased dopamine release in the No WD ethanol consuming subjects relative to controls. In agreement, we also found a trend towards a decreased DOPAC/DA ratio, suggesting greater dopamine utilization or turnover, in different subjects from the same treatment group.

The discrepancies between previously published work and the work presented here could be accounted for by differences in ethanol treatment (CIE vs. two bottle choice) or brain region differences (NAc vs. DS). Similarly, studies in macaques have also found varying effects of long-term ethanol consumption on evoked dopamine release. For example, one study in cynomolgus macaques [8] found an increase in caudate dopamine uptake rate and no change in dopamine release. A separate study in NAc found no change in dopamine release but an increase in DA uptake [11]. In another cynomolgus macaque study in the NAc found increased dopamine release and uptake and decreased dopamine release and uptake in the dorsolateral caudate [10]. In a previous study [9], we found sex- and withdrawal status-dependent changes in dopamine release and uptake in caudate and putamen.

### 3.3. Altered Presynaptic Regulation of Dopamine Release

While we did not note any significant group differences in evoked dopamine release, we did observe an increase in the ability of D2 dopamine receptors to inhibit dopamine release in the dorsal striatum. Similar findings have been reported in the NAc of mice and rhesus macaques after chronic intermittent ethanol vapor exposure [16] or long-term ethanol self-administration [11], as well as in the caudate nucleus of long-term alcohol self-administering cynomolgus macaques [8]. However, one study found no change D2 dopamine autoreceptor sensitivity following chronic ethanol treatment [17]. In contrast, in a previous study, we found a sex-dependent decrease in D2 dopamine autoreceptor function following long-term ethanol self-administration [9]. The differences in these studies could be attributed to several factors including species differences (i.e., C57BL6J vs. cynomolgus macaque vs. rhesus macaque), brain region differences, or ethanol treatment. It is also noteworthy that cholinergic interneurons in the striatum express D2 dopamine receptors. Thus, application of quinpirole might also inhibit acetylcholine release from these neurons which might in turn inhibit dopamine release. This possibility will be assessed in future studies.

We also examined the β2-containing nAChR contribution to evoked dopamine release in all groups. Given the lack of a significant role for α7 nAChRs in modulating dopamine release, we used DHβE, which has been shown to be selective for β2-containing nAChRs. One day of two-bottle choice drinking had no significant effect on the ability of nAChR blockade to decrease dopamine release. However, following one month of two-bottle choice drinking, the nAChR contribution to evoked dopamine release was decreased, suggesting a cholinergic hypofunctional state. We previously reported a similar finding in rhesus macaques [9]. This suggests a cross species conserved effect of chronic alcohol consumption that may extend to humans. Importantly, this effect may also be conserved between sexes as the nAChR antagonist, DHβE, had no significant effect on dopamine release in the putamen of long-term ethanol self-administering female rhesus macaques [9]. Future work will determine the locus of this putative cholinergic hypofunction. For example, decreases in presynaptic nAChRs on dopamine axons or changes in nAChR subunit composition could contribute to the decreased contribution of acetylcholine to dopamine release. Alternatively, the chronic ethanol induced deficit in cholinergic signaling could also be due to decreases in the presynaptic release of acetylcholine or loss of cholinergic neurons. Indeed, our previous study suggested a decrease in acetylcholine release and not a loss of cholinergic neurons in the caudate and putamen. This possibility is also supported by the modest success of varenicline, a partial nAChR agonist, in the treatment of AUD [30] and which may exert its beneficial effects by compensating for a chronic alcohol induced cholinergic circuit hypofunction.

## 4. Materials and Methods

### 4.1. Subjects

Male C57BL/6J mice were obtained from The Jackson Laboratory (strain ID: 000664) at 10 weeks old and housed two per cage. After a one-week acclimation period, the mice were individually housed in Techniplast cages with two bottle spots and two bottles, each containing tap water. Mice were given an additional week to acclimate to drinking from two bottles. Then, mice were weighed, assigned to a treatment group (balanced by average initial body weight), and two-bottle choice ethanol consumption was assessed as described below. All experimental procedures were approved by the National Institute on Alcohol Abuse and Alcoholism Animal Care and Use Committee and were performed in accordance with National Institutes of Health guidelines. Food and water were available *ad libitum* throughout the study.

### 4.2. Experimental Design

To assess the effects of acute and chronic ethanol consumption on dorsal striatal dopamine release, we exposed subjects to either one day (acute) or one month (chronic) of two-bottle choice alcohol consumption. To assess the effects of withdrawal, another group of mice underwent one month of two-bottle choice ethanol consumption and one week of withdrawal. After each subject completed their treatment, they were used for FSCV or dopamine tissue content experiments.

### 4.3. Two-Bottle Choice Ethanol Consumption

Beginning at 12 weeks, one of the water bottles was replaced with a similar bottle containing a 10% ethanol solution. Thereafter, the water and ethanol bottles were weighed three times per week to determine water and ethanol consumption. The bottle positions were alternated at each weighing to avoid a bottle position preference. Mice were weighed twice per week throughout the experiment. Ethanol consumption was determined (as g/kg of body weight) for each subject three times per week. Additionally, lifetime ethanol intake was tabulated for the chronic alcohol groups. Empty cages with two control tubes (one with water and the other with the ethanol solution) were used to account for any evaporation or spillage. Preference was determined by dividing the volume of ethanol solution consumed by the total volume of ethanol and water consumed. Total liquid intake was also assessed for each subject.

### 4.4. Blood Ethanol Concentration

In a separate cohort of mice treated identically to the chronic two bottle choice groups, blood ethanol concentrations were determined. Mice were allowed to drink normally except one day before the end of the month-long drinking period or after one week of withdrawal, tail vein blood samples were collected three hours into the dark cycle when mice consume greater amounts of ethanol [31,32]. The tail bloods were collected into heparinized capillary tubes and sealed with parafilm while all blood samples were collected (<1 h). The capillary tubes were then centrifuged in a hematocrit centrifuge at 10,000 rpm for 5 min and the plasma was used to determine blood ethanol concentration using the Pointe Scientific Alcohol Reagent Kit according to the manufacturer’s protocol and a SpectraMax 190 Absorbance Plate Reader (Molecular Devices).

### 4.5. Tissue Dopamine Content

In a separate cohort of mice treated identically to the chronic two bottle choice groups, striatal tissue samples were collected for dopamine and DOPAC tissue level analysis with HPLC with electrochemical detection [33]. Immediately after mice completed their drinking treatment, they were anesthetized with isoflurane and rapidly decapitated. Brains were extracted on ice and snap frozen in a dry ice-isopentane mixture. The frozen brains were placed in a brain matrix and 1mm thick coronal sections were prepared on dry ice. One millimeter diameter tissue punches were collected from the dorsal striatum of both hemispheres of two consecutive coronal sections. The tissue punches were weighed and placed in a 0.4 M perchloric acid/0.5 mM EDTA solution for homogenization on ice. The homogenates were then centrifuged at 12,000× *g* for 10 min and the supernatants collected in new tubes for dopamine and DOPAC determination. The samples were stored at −80 °C until use.

### 4.6. Brain slice Preparation

Mice were deeply anesthetized with isoflurane and rapidly decapitated. The brains were rapidly extracted on ice and immediately immersed in ice-cold, carbogen-saturated (95% O_2_/5% CO_2_), cutting ACSF containing the following (in mM): 194 Sucrose, 30 NaCl, 4.5 KCl, 26 NaHCO_3_, 1.2 NaH_2_PO_4_, 10 dextrose, and 1 MgCl_2_. After one minute, the brain was removed from the solution and the cerebellum was removed to create a flat surface. The blocked off brain surface was placed on a Kimwipe briefly and then placed onto cyanoacrylate adhesive on a Leica VT1200S specimen disk. The specimen disk was them moved into the VT1200S buffer tray filled with ice-cold, carbogen-saturated cutting ACSF. The VT1200S vibratome was then used to prepare 300 µm thick coronal sections containing the dorsal striatum. The slices were hemisected, then incubated for 30 min at 32 °C in voltammetry ACSF containing (in mM): 126 NaCl, 2.5 KCl, 26 NaHCO_3_, 1.2 NaH_2_PO_4_, 10 dextrose, 20 HEPES, 2.4 CaCl_2_, 1.2 MgCl_2_, and 0.4 L-ascorbic acid, pH 7.4. Then, the slices were moved to room temperature and incubated for at least one hour in the same solution before beginning FSCV experiments.

### 4.7. Fast-Scan Cyclic Voltammetry

Fast-scan cyclic voltammetry experiments were performed as previously described [9,34]. Briefly, striatal slices were moved to the rig and allowed to acclimate to the slice recording chamber for at least 10 min before beginning experiments. Voltammetry ACSF was continuously perfused on the slice at a rate of 1.5–2 mLs/min and at a temperature of 32 °C. Once ready a twisted bipolar stainless steel stimulating electrode was lowered into the dorsal striatum using a Narishige manual micromanipulator under a Zeiss StereoDiscovery V8 microscope. Then, a carbon fiber electrode (CFE) was placed into the slice approximately 300 µm from the stimulating electrode as shown in Figure 1B. Cyclic voltammograms were generated by applying a triangle waveform from −0.4 V to + 1.2 V to −0.4 V at a scan rate of 400 V/s. Cyclic voltammograms were generated at a rate of 10 Hz using a Chem-Clamp potentiostat (DAGAN) and DEMON Voltammetry and Analysis software [35]. Dopamine release was evoked by application of a single electric stimulation (1 ms pulse width) generated by a DS3 constant current stimulus isolator (Digi Timer) at varying stimulation intensities every three minutes. The stimulation intensity was determined from the stimulation intensity input-output curve for each slice. A stimulation intensity evoking a 40–60% maximal dopamine response was subsequently used for each experiment. Once five stable consecutive responses were obtained, experiments could begin.

For experiments examining D2 dopamine autoreceptor regulation of dopamine release, the D2-like dopamine receptor agonist, quinpirole (30 nM), was bath applied to slices for 30 min. For experiments examining the cholinergic contribution to dopamine release, the β2-containing nAChR antagonist, DHβE (1 µM), was bath applied to slices for 15 min. For the variable stimulation frequency experiments, burst stimulations consisted of 6, 1 ms stimulations at either 10, 50, or 100 Hz, applied every 5 min in a random order and at least two replicates at each frequency. At the end of each recording day, CFEs were calibrated against a 1 µM dopamine standard.

Carbon fiber electrodes were fabricated in house by aspirating a single, 7 µm diameter, carbon fiber strand (T650 fiber; Goodfellow) through a 1.2 mm OD borsilicate glass capillary tube (A-M Systems). The glass capillary tube was then placed in a PE-22 vertical puller (Narishige) and pulled to encase the carbon fiber in glass on one end. The pulled CFE was trimmed under 100X total magnification to a final exposed carbon fiber length between 100–150 µm past the glass seal.

### 4.8. Drugs and Reagents

Dopamine-HCl and ethanol were obtained from Sigma-Aldrich. Quinpirole and DHβE were obtained from Tocris Bioscience. All drugs were dissolved in voltammetry ACSF. All other reagents were obtained from Sigma-Aldrich.

### 4.9. Statistics

All data were entered and organized in Google Sheets before being exported to GraphPad Prism 8 software for statistical analysis and graphing. Unpaired *t*-tests were used to compare lifetime ethanol intake and BEC data. Two-way ANOVA was used to analyze ethanol consumption, preference, liquid intake, body weight data, input-output curves, DOPAC/DA ratios, and quinpirole data. Three-way ANOVA 3 factor mixed-effects analysis was used for the DHβE and train stimulation data.

## Data Availability

The data that support the findings of this study are available from the corresponding authors upon reasonable request.
